# The Typhoid Fever Surveillance in Africa Program: Geospatial Sampling Frames for Household-based Studies: Lessons Learned From a Multicountry Surveillance Network in Senegal, South Africa, and Sudan

**DOI:** 10.1093/cid/ciz755

**Published:** 2019-10-30

**Authors:** Stephen Baker, Mohammad Ali, Jessica Fung Deerin, Muna Ahmed Eltayeb, Ligia Maria Cruz Espinoza, Nagla Gasmelseed, Justin Im, Ursula Panzner, Vera V Kalckreuth, Karen H Keddy, Gi Deok Pak, Jin Kyung Park, Se Eun Park, Arvinda Sooka, Amy Gassama Sow, Adama Tall, Stephen Luby, Christian G Meyer, Florian Marks

**Affiliations:** 1 Oxford University Clinical Research Unit, Ho Chi Minh City, Vietnam; 2 Department of Medicine, University of Cambridge, United Kingdom; 3 Johns Hopkins University, Baltimore, Maryland; 4 International Vaccine Institute, Seoul, Republic of Korea; 5 Faculty of Medicine at the University of Gezira, Wad-Medani, Sudan; 6 Faculty of Science, University of Hafr Al Batin, Saudi Arabia; 7 Faculty of Health Sciences, University of the Witwatersrand; 8 National Institute for Communicable Diseases, Johannesburg, South Africa; 9 Institut Pasteur de Dakar, Senegal; 10 Université Cheikh Anta Diop de Dakar, Senegal; 11 Infectious Diseases and Geographic Medicine, Stanford University, California; 12 Institute of Tropical Medicine, Eberhard Karls University, Tübingen, Germany; 13 Duy Tan University, Da Nang, Vietnam

**Keywords:** satellite imagery, geospatial sampling frame, positional accuracy, sub-Saharan Africa

## Abstract

**Background:**

Robust household sampling, commonly applied for population-based investigations, requires sampling frames or household lists to minimize selection bias. We have applied Google Earth Pro satellite imagery to constitute structure-based sampling frames at sites in Pikine, Senegal; Pietermaritzburg, South Africa; and Wad-Medani, Sudan. Here we present our experiences in using this approach and findings from assessing its applicability by determining positional accuracy.

**Methods:**

Printouts of satellite imagery combined with Global Positioning System receivers were used to locate and to verify the locations of sample structures (simple random selection; weighted-stratified sampling). Positional accuracy was assessed by study site and administrative subareas by calculating normalized distances (meters) between coordinates taken from the sampling frame and on the ground using receivers. A higher accuracy in conjunction with smaller distances was assumed. Kruskal-Wallis and Dunn multiple pairwise comparisons were performed to evaluate positional accuracy by setting and by individual surveyor in Pietermaritzburg.

**Results:**

The median normalized distances and interquartile ranges were 0.05 and 0.03–0.08 in Pikine, 0.09 and 0.05–0.19 in Pietermaritzburg, and 0.05 and 0.00–0.10 in Wad-Medani, respectively. Root mean square errors were 0.08 in Pikine, 0.42 in Pietermaritzburg, and 0.17 in Wad-Medani. Kruskal-Wallis and Dunn comparisons indicated significant differences by low- and high-density setting and interviewers who performed the presented approach with high accuracy compared to interviewers with poor accuracy.

**Conclusions:**

The geospatial approach presented minimizes systematic errors and increases robustness and representativeness of a sample. However, the findings imply that this approach may not be applicable at all sites and settings; its success also depends on skills of surveyors working with aerial data. Methodological modifications are required, especially for resource-challenged sites that may be affected by constraints in data availability and area size.

Household sampling is a common and resource-efficient method for rapid, in-depth investigations in population-based studies. This includes assessment of health indicators, morbidity and mortality rates, vaccination coverage, healthcare behavior, and relevant sociodemographic, socioeconomic, and ecological information among the population of interest [1–3]. Data derived from a representative sample allow inference findings to an entire population under investigation [1–4]. A comprehensive sampling frame or household list largely reduces the possibility of selection bias. Ideally, a sampling frame is available through a demographic surveillance system (DSS), which longitudinally records demographic and vital statistics of individuals [5, 6]. However, DSSs are limited to distinct sites and their implementation and maintenance require long-term planning and enduring financial support [6].

Sampling procedures in resource-limited settings inherently are affected by distinct drawbacks, such as scarcity of demographic and geographic data and lack of a comprehensive sampling frame. There are, however, several options to cope with the absence of a household list by using convenience samples [7], choosing a random starting point, and selecting sampling units by applying systematic sampling [8–10]. Moreover, chain or respondent-driven sampling may be applied [11, 12]. In such a design, study subjects recruit additional subjects until a desired sample size is achieved. Further approaches include segment sampling, where subunits in a study area are divided into segments and all sampling units of a selected segment are enrolled [13], mosaic formation or rasterizing with continuous geographic data of a study area transformed into a raster sampling frame from which sampling units are drawn [14], or overlaying study areas with grid cells to establish a sampling frame [15–17]. Geographical information systems (GISs) are also used if household lists are not available [2, 4, 18]. Kondo et al [2] have described spatial sampling with modifications for postdisaster scenarios and ecological, environmental, and social studies. Satellite imagery has been applied to generate sampling frames for research on mosquito-borne diseases [19] and the impact resulting from natural and humanitarian crises in addition to healthcare behavior [20].

We have applied satellite imagery to establish sampling frames to assess healthcare behavior at three resource-limited sites (Table 1) of the Typhoid Fever Surveillance in Africa Program (TSAP) [21]. The TSAP network investigated the incidence of *Salmonella* infections at thirteen sentinel sites in ten countries (Burkina Faso, Ethiopia, Ghana, Guinea-Bissau, Kenya, Madagascar, Senegal, South Africa, Sudan, Tanzania) during the period 2010–2014 [22]. Satellite maps combined with Global Positioning System (GPS) receivers were used to locate and to verify the locations of selected structures on the ground. We present our experiences of this approach and findings from assessing its applicability by determining positional accuracy. Findings on the assessment of healthcare behavior within the TSAP study were presented elsewhere [23].

**Table 1.** Baseline Characteristics by Study Site

Country	Senegal			South Africa						Sudan						
Site	Pikine			Pietermaritzburg						Wad-Medani						
Study period	September 2012–January 2013			September–December 2013						August–September 2013						
Google Earth Pro																
Date of satellite imagery update(s)	March, June, October, November, December 2012			March, May, July 2013						March, September, October 2013						
Date satellite imagery used	June 2012			July 2013						March 2013						
Elapsed time between imagery used and study conduct	2–3 mo			1–2 mo						4–5 mo						
Setting	Urban to semiurban			Urban to semiurban						Urban to semiurban						
Area, km^2^	7.98			343.56						6.47						
Administrative subunits	6			22						10						
Population	342 178 (2012: [22–24, 30])			361 582 (2011: [27–29])						48 000 (2012: [25, 26, 33])						
Population density per km^2^	42 886			1052						7409						
Population density per km^2^, by administrative subunit	AdSub	Density	AdSub	Density	AdSub	Density	AdSub	Density	AdSub	Density	AdSub	Density	AdSub	Density	AdSub	Density
	1	75 999	4	49 361	1	1386	9	445	17	4292	1	22 848	5	2994	9	2727
	…	…	…	…	2	1070	10	3634	18	6875	…	…	…	…	…	…
	…	…	…	…	3	423	11	662	19	1960	2	15 810	6	12 370	10	26 293
	2	60 800	5	28 217	4	409	12	2298	20	6147	…	…	…	…	…	…
	…	…	…	…	5	448	13	3472	21	4823	3	81 370	7	11 425	…	…
	…	…	…	…	6	670	14	1203	22	2246	…	…	…	…	…	…
	3	18 000	6	44 081	7	536	15	4607	…	…	4	10 218	8	1911	…	…
	…	…	…	…	8	677	16	6049	…	…	…	…	…	…	…	…
No. of enumerated structures	45 510			100 439						32 905						
Structure density per km^2^	5829			292						5086						
Structure density per km^2^, by administrative subunit	AdSub	Density	AdSub	Density	AdSub	Density	AdSub	Density	AdSub	Density	AdSub	Density	AdSub	Density	AdSub	Density
	1	2087	4	5675	1	385	9	124	17	1192	1	2195	5	3183	9	9096
	…	…	…	…	2	297	10	1009	18	1910	…	…	…	…	…	…
	…	…	…	…	3	117	11	184	19	544	2	4409	6	998	10	8158
	2	3207	5	5197	4	114	12	638	20	1708	…	…	…	…	…	…
	…	…	…	…	5	125	13	964	21	1340	3	3833	7	4707	…	…
	…	…	…	…	6	186	14	334	22	624	…	…	…	…	…	…
	3	2406	6	11 591	7	149	15	1280	…	…	4	2096	8	6072	…	…
	…	…	…	…	8	188	16	1680	…	…	…	…	…	…	…	…

Abbreviation: AdSub, administrative subunit.

## MATERIALS AND METHODS

The study was implemented at sites in Pikine, Senegal (September 2012–January 2013), Pietermaritzburg, South Africa (September–December 2013), and Wad-Medani, Sudan (August–September 2013). It was approved by the ethics committees of the collaborating institutions and the ethical review board of the International Vaccine Institute.

### Study Sites

Details of the selection of study sites have been described previously [21, 23]. In brief, the sites were chosen based on reports on human *Salmonella* infections, an infrastructure suitable for the surveillance of acute febrile conditions, and access to healthcare [22]. Pikine is a semiurban region in the east of Senegal’s capital, Dakar. Pietermaritzburg is the capital of the KwaZulu-Natal Province and located at the southeastern coast of South Africa. Wad-Medani is southeast of Sudan’s capital Khartoum and the capital of the Al Jazirah State.

Sites were highly diverse with respect to population and study area sizes, including administrative subunits, or AdSubs (Table 1). They varied in topography and vegetation, the road network, and formal and informal settlements (Supplementary Figure 1*A–C*). Across all sites, single-story and single-family households were common compared to multistory and multifamily households. A household was defined as a person or a group of related or unrelated persons living in the same dwelling unit, acknowledging one adult individual as household head, sharing the same housekeeping arrangements, and independently procuring food and other essentials for living [23].

Population sizes and boundaries of each site and its AdSubs were determined by combining different sources. Among them were up-to-date demographic information, population summary figures and growth rates [24–32], records of healthcare facilities [23], and administrative and geographic data, including sketch-maps [31, 33–37]. Boundaries of a study area and its AdSubs were transferred from sketch maps, or the sketch maps were digitized and superimposed onto Google Earth Pro imagery (version 6.2.2.6613; Google, Mountain View, California).

### Sampling Frame

**Figure 1.**
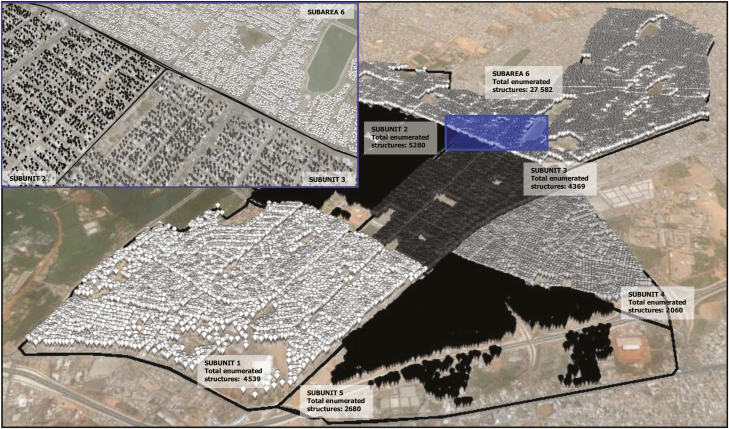
Sampling frame of the study area and the administrative subunits (AdSubs) in Pikine, Senegal. Different colors depict the structures belonging to each AdSub. Illustration top left: enlarged illustration of enumerated structures for subunits 2, 3, and 6 (blue highlighted rectangle in main figure).

We used the latest satellite imagery available from Google Earth Pro (Pikine: June 2012; Pietermaritzburg: July 2013; Wad-Medani: March 2013; Table 1). Every single-standing structure defined as an edifice not connected to another structure in the respective satellite image, of appropriate size and rectangular or square shape was enumerated by positioning a placemark at its approximate center. Irregularities in size, shape, and spacing between structures made manual enumeration preferable (Supplementary Figure 1*A–C*). Buildings of nonresidential character based on best local knowledge were not enumerated. Figure 1 shows the sampling frame in Pikine. Sampling frame data were exported from Google Earth Pro and imported into ArcGIS version 10.2 (Esri, Redlands, California) to assign a unique identifier and coordinates to each placemark.

### Random Sample Selection

**Figure 2.**
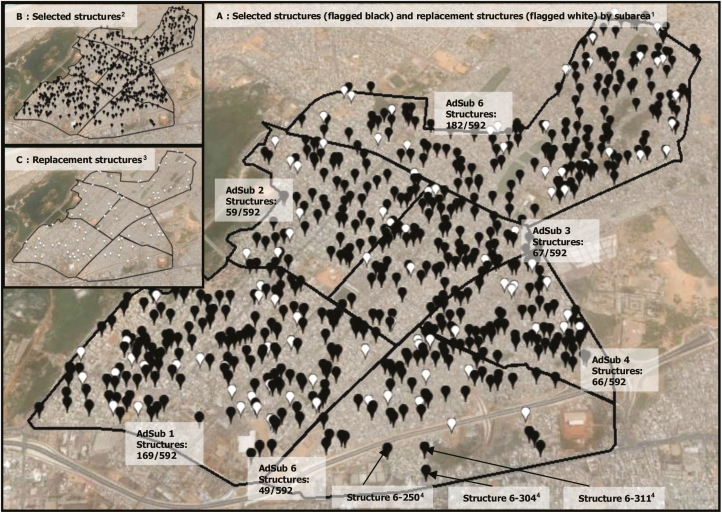
Weighted-stratified random sampling of structures in Pikine, Senegal. ^1^Selected structures (N_0_) as per sample size calculation for the total survey area and each administrative subunit (AdSub) (flagged black) and replacement structures for the total survey area and each AdSub (flagged white). ^2^Selected structures (N_0_) for the total survey area and each AdSub (flagged black). ^3^Replacement structures for the total survey area and each AdSub (flagged white). ^4^Identifiers (6–250, 6–304, 6–311) and the geographic coordinates (6–250: N14°44.702′/ W17°23.408′; 6–304: N14°44.632′/W17°23.284′; 6–311: N14°44.708′/W17°23.289′) obtained from Google Earth Pro.

The minimum number of household interviews required (N_0_) was 492 at each site. Sample size calculations were based on the Cochran formula for categorical data; their assumptions (95% confidence interval, 5% precision, 2.0 design effect) have been described previously [23]. N_0_ was distributed by applying weighted-stratified sampling and selected from a sampling frame by serial simple random selection using Microsoft Visual FoxPro (version 9.0; Microsoft, Redmond, Washington; Figure 2). Approximately 10%–20% replacement structures by AdSub (Figure 2) were chosen using the same selection procedure as for N_0_ if a sample structure was a nonresidential building, eligible respondents were not available after three consecutive visits, or participation was refused.

### Structure Identification

The study teams were comprised of community nurses and healthcare workers experienced in conducting household surveys (Pikine: eight interviewers; Wad-Medani: eight interviewers; Pietermaritzburg: twenty interviewers). They were trained on all study procedures and, in particular, on the localization and the verification of locations of selected structures by using satellite maps and GPS receivers as not all interviewers were familiar with observing study areas from an aerial perspective. The assignment of AdSubs and sample structures to the surveyors was done arbitrarily across all sites.

Poster-sized (60 × 60 cm to 60 × 90 cm) printouts of Google Earth Pro satellite imagery with high resolution (approximately 500–600 m “eye altitude” in high-density and approximately 1700–1800 m “eye altitude” in low-density settings; Table 1 and Supplementary Figure 1*A–C*) were prepared to depict selected structures flagged and labeled with the respective identifiers (Figure 2). “Eye altitude” is a term used in Google Earth for viewing heights. GPS receivers (Garmin-eTrex, GPS accuracy <10 m; differential GPS [Wide Area Augmentation System], accuracy 3 m; 12-channel receiver; Garmin Ltd, Lenexa, Kansas) set in World Geodetic System 1984 were used to verify locations of sample points. Once a structure was identified on the ground with reference to a landmark, interviewers took the GPS readings allowing sufficient time to obtain satellite signals with an accuracy of ≤ three meters. The GPS receiver was positioned to the structure as close as possible, in a static position, and an open area, strictly avoiding tree cover, roof cover, balconies, or verandas to ensure barrier-free reading, reduction of interferences, and increased accuracy. A structure was replaced if it could not be identified correctly.

### Positional Accuracy

We have evaluated the applicability of the described approach by determining the positional accuracy of sample points. This was performed by analyzing distances (meters) between geographic coordinates taken from the sampling frame and on the ground by receivers, assuming zero distances and approximation of earths radius based on the Pythagorean theorem [7]. We surmised that the smaller the distances, the more accurately structures were identified and, thereby, the more representative were our samples by AdSubs and sites. The computed distances were normalized for improved comparability (Supplementary Table 1). Microsoft Office Excel version 2010, expanded with Excel add-on tools, was applied for all calculations. The equations are explained in Supplementary Table 1.

Distances by each site and AdSub were assessed by generating medians, interquartile ranges (IQRs), and quartiles displayed by box plots, and root mean square errors (RMSEs) [38, 39]. Obtained distances were categorized into quintiles and graded correspondingly as very good (lowest quintile), good, fair, poor, and very poor (highest quintile). Furthermore, distances by AdSub of Pietermaritzburg only were classified into tertiles based on population and structure density (Supplementary Figure 2) and graded as low, medium, and high; medians, IQR, and quartiles by grade or setting are given in box plots. The positional accuracy of the described approach was also assessed for each interviewer of Pietermaritzburg by computing medians, IQRs, and quartiles displayed by box plots, RMSEs, and quintiles of distances, followed by grading. In addition, the nonparametric rank-based Kruskal-Wallis test was performed to evaluate distance differences by setting and interviewer of Pietermaritzburg. The test was followed by Dunn multiple pairwise comparisons, assuming rejection of the null hypothesis (H_0_) of no difference in the distribution of distances by setting or interviewer. Bonferroni correction was applied to compensate for incorrectly rejecting the H_0_ [40, 41].

## RESULTS

### Sampled Structures

The number of enumerated structures constituting the sampling frame was 45 510 in Pikine (Figure 1), 100 439 in Pietermaritzburg, and 32 905 in Wad-Medani. The enumeration and preparation of the satellite maps, including random selection and visualization of selected sample points using Google Earth Pro, took approximately two weeks per site.

In Pikine, 597 structures were included into the study, of which 495 (83%) were initially selected and 101 (17%) were substituted. In Pietermaritzburg, 2402 sample points were identified of which 461 (19%) were replaced; of those replaced, 164 (36%) were nonresidential, 143 (31%) were excluded due to refusal in participation, and in 154 (33%) household members were not addressable after 3 consecutive visits. A total of 549 structures were enrolled in Wad-Medani, of which 412 (75%) were initially selected and 137 (25%) were replaced.

### Accuracy of Sample Identification

**Figure 3.**
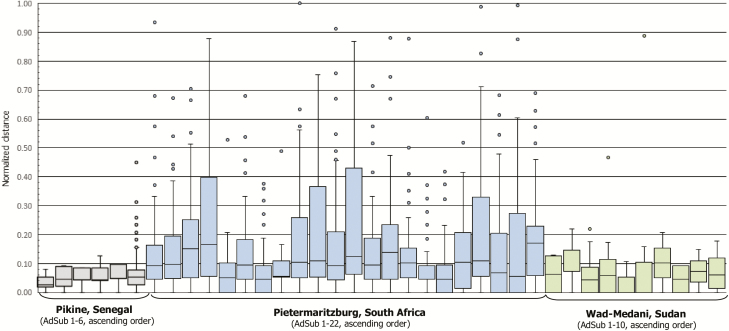
Normalized distances by administrative subunit (AdSub) in Pikine (Senegal), Pietermaritzburg (South Africa), and Wad-Medani (Sudan). Each individual box plot shows the range of normalized distances indicated as vertical line; bottom whisker (minimum normalized distance to first quartile; non-outlier), first quartile (25% of normalized distances/25th percentile), second quartile or median (50% of normalized distances/50th percentile), third quartile (75% of normalized distances/75th percentile), top whisker (third quartile to maximum normalized distance; non-outlier), and outliers plotted as circles. Senegal: The root mean square error (RMSE) of normalized distances by AdSub was 0.04, 0.06, 0.06, 0.06, 0.07, and 0.13 (ascending order). South Africa: The RMSE of normalized distances by AdSub was 0.32, 0.21, 0.40, 0.63, 0.29, 0.25, 0.32, 0.16, 0.32, 0.98, 0.56, 0.69, 0.28, 0.35, 0.38, 0.22, 0.10, 0.41, 0.35, 0.22, 0.54, and 0.31 (ascending order). Sudan: The RMSE of normalized distances by AdSub was 0.08, 0.11, 0.22, 0.10, 0.06, 0.43, 0.11, 0.06, 0.07, and 0.09 (ascending order).

**Figure 4.**
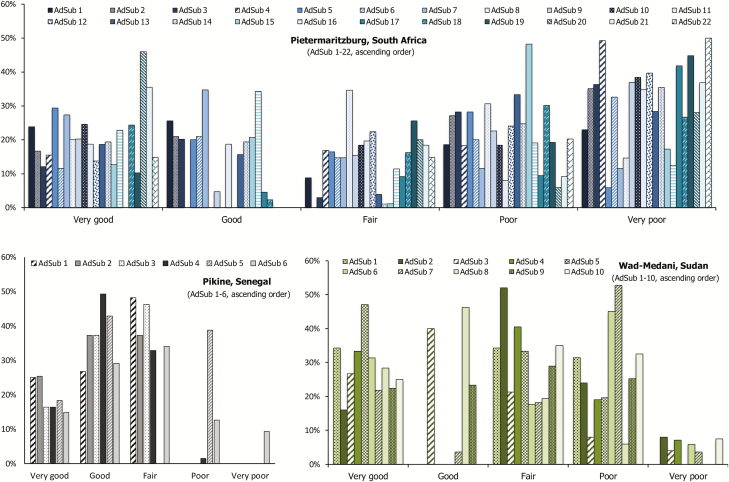
Normalized distances (meters) categorized into quintiles and graded accordingly by administrative subunit (AdSub) of each site. Each bar shows the frequency of normalized distances categorized into quintiles by AdSub and graded correspondingly as very good (lowest quintile), good, fair, poor, and very poor (highest quintile). Senegal: very good, 19.4%; good, 33.6%; fair, 36.8%; poor, 7.3%; and very poor, 2.9%. South Africa: very good, 20.4%; good, 14.6%; fair, 12.9%; poor, 22.2%; and very poor, 29.9%. Sudan: very good, 28.3%; good, 16.1%; fair, 27.7%; poor, 25.0%; and very poor, 2.9%.

The median normalized distances (formula, refer to Supplementary Table 1) and RMSEs were 0.05 (IQR, 0.03–0.08) and 0.08, respectively, in Pikine; 0.09 (IQR, 0.05–0.19) and 0.42 in Pietermaritzburg; and 0.05 (IQR, 0.00–0.10) and 0.17 in Wad-Medani. Quartiles of distances by AdSub of each site, including RMSEs, are displayed in Figure 3. The quintile categorization by site and AdSub revealed largest proportions in the categories “good” and “fair” in Pikine, “poor” and “very poor” in Pietermaritzburg, and “very good” and “fair” in Wad-Medani (Figure 4); 4 of Pietermaritzburg’s AdSubs [5, 7, 16, 17] with the largest proportions in the categories “very good” and “good” showed also low median values, IQRs, and RMSEs.

The median distances and RMSEs were 0.09 (IQR, 0.05–0.18) and 0.40 in the low-density, 0.11 (IQR, 0.05–0.23) and 0.37 in the medium-density, and 0.09 (IQR 0.00–0.15) and 0.47 in the high-density setting of Pietermaritzburg; quartiles of distances by AdSub and setting are illustrated in Supplementary Figure 2. The quintile categorization revealed largest proportions in the categories “poor” and “very poor” in the low- and medium-density settings, and “very good” and “poor” in the high-density setting. An H_c_ (2) = 5.991 with *P* < .0001 (H_c_observational_ = 28.202 ≥ H_c_critical_ = 5.991; rejection of H_0_) was found by using Kruskal-Wallis test. The two-tailed *P* values of Dunn multiple pairwise comparisons (Bonferroni significance level = .0167) indicated significant differences between the low- and medium-density settings (*P* = .0007) as well as the medium- and high-density (*P* = .0001) settings.

Quartiles of distances by interviewers of Pietermaritzburg are displayed in Supplementary Figure 3, including the RMSEs. The quintile categorization is depicted in Supplementary Figure 4. Largest proportions were found in the categories “very good” and “good” for one-third of the interviewers (interviewers 3, 6, 8, 10, 15, and 16) in addition to small median values, narrow IQRs, and low RMSEs. In contrast, the largest proportions were seen in the categories “poor” and “very poor” for one-fifth of interviewers (interviewers 5, 14, 18, and 19) besides large median values, wide IQRs, and high RMSEs. The Kruskal-Wallis test revealed an H_c_ (19) = 30.144 with *P* < .0001 (H_c_observational_ = 239.317 ≥ H_c_critical_ = 30.144; rejection of H_0_); *P* values of Dunn comparisons by interviewer are given in Supplementary Table 2.

## DISCUSSION

The application of satellite imagery is an efficient tool as also shown by other research conducted, even for sampling frames of varying sizes and in diverse sites as seen in our multicountry study. While we have chosen this particular approach for conducting household surveys in our TSAP study sites, we did not conduct a head-to-head comparison to other methods and, hence, can only report here on the experiences we made. With regard to its applicability we observed here, the evaluation of the described geospatial approach revealed a lower accuracy for Pietermaritzburg and a higher accuracy for Pikine and Wad-Medani in the identification of structures. This finding is based on largest median values, IQRs, RMSEs, and outliers of distances (Figure 3), as well as greatest proportions in the high and highest quintile categories (Figure 4) observed for Pietermaritzburg.

Though the analyses revealed only minor discrepancies in the statistical parameters (median, IQR, RMSE, outliers, quintile categorization) by setting of Pietermaritzburg, Kruskal-Wallis and Dunn tests indicated significant differences in distances between low- and medium-density and medium- and high-density settings. This suggests that the approach presented could be followed more accurately in the low- and high-density settings, supporting findings of previous studies [2]. Moreover, this result may explain why the geospatial method was more successful in Pikine and Wad-Medani, both settings of high population density (Table 1). However, further investigations are required to assess if this is a real finding or just a result caused by an almost one-third smaller sample size in the medium-density setting of Pietermaritzburg. Future investigations should also include thorough research on drivers of accuracy. However, since we performed a retrospective assessment of our geospatial approach, this is not available, which is a major limitation.

Interviewers in Pietermaritzburg performed the presented approach with varying exactness, corroborated by the interrelation observed between the quintile categorization, medians, IQRs, and RMSEs by interviewers. For interviewers yielding the largest proportions in the lowest and low quintiles, small median values, IQRs, and RMSEs were observed. In contrast, if largest proportions were found in the high and highest quintiles, large median values and wide IQRs and high RMSEs were seen. Kruskal-Wallis and Dunn tests revealed statistically significant differences for those interviewers showing high accuracy compared to interviewers with poor accuracy in carrying out this approach and vice versa. However, we cannot provide a plausible explanation for this finding. A much larger team of interviewers might have resulted in a general poorer success.

A limitation is that we could not directly compare our rigorous method with alternatives as we have no quantifiable data on whether the method utilized in the TSAP program justifies the extra work and cost compared to other methods. A pitfall of satellite imagery is the lack of structure differentiation into residential or nonresidential from the aerial perspective. In fact, this applied to all sites [2, 4, 20]. The need of replacing structures may increase the sample size and prolong the study conduct. Incorrect identification of terrestrial sample points is a further weakness that applied to all sites, in particular in areas where buildings were not lined up as described in an earlier study [2], clustered, or interlaced. The latter problems were likely caused by interfering factors like reflective materials (water, metallic objects), obstructive buildings, and environmental diversity influencing the accuracy of GPS readings, and resulting in positional errors (multipath effect) [7, 38, 39, 42]. A further limitation is that there may have been some degree of inaccuracy in obtaining GPS readings of selected sample points in the field due to the intrinsic inaccuracy, which we have not adjusted for in the analysis. Structures were enumerated by positioning a placemark at the approximate center, whereas GPS readings on the ground were performed simply as close as possible to structures.

Nevertheless, the use of satellite maps was beneficial across all sites. They guided interviewers on the ground, facilitated identification of structures, and allowed the recording of the daily study progress. This is in accordance with previous observations [20, 43]. Geospatial approaches relying on computerized random selection of sample points was applied across all three sites. This assures that each point has the same probability of being chosen and thus increases the degree of randomness of samples selected. It also minimizes the possibility of introducing selection bias by study staff by allowing a prioritized sample selection in densely inhabited areas, or near a study area’s center or random starting point for instance, and a nonprioritized selection of structures in remote areas [2, 7]. We believe that this makes spatial sampling frames superior to other techniques that use no or inappropriate sampling frames such as convenience sampling [7], systematic sampling [8, 9] or chain or respondent-driven sampling [11, 12], and which rely on a homogeneous study population as applied in segment sampling [13]. An advantage of manual enumeration as performed across our sites overautomated techniques such as mosaic formation, rasterizing [14], or grid cells [15–17] is that it is particularly suitable if buildings are irregular in size, shape, and spacing.

## CONCLUSIONS

The application of satellite imagery offers a broad spectrum for research and has been deployed in the TSAP program. Actual healthcare utilization data are part of the manuscripts that were published previously [22, 23]. The evaluation of this approach conducted in a comparable and standardized manner indicates that sources of selection bias are reduced and robustness is increased, if performed with high accuracy. However, our findings imply that the applicability of this geospatial approach may not be suitable for all sites and settings, in particular not for medium-density settings due to an overall poorer study success. They also indicate that the skills of staff working with aerial data considerably affect the outcome of this approach, as seen for Pietermaritzburg. A potential modification of the method may be to apply ArcGIS tools to randomly spread geographic points across study areas based on a required sample size and the sampling strategy instead of enumerating individual structures. This needs to be examined in other resource-limited sites that are even more affected by data availability and constraints in area size than those selected for the present study.

## Supplementary Data

Supplementary materials are available at *Clinical Infectious Diseases* online. Consisting of data provided by the authors to benefit the reader, the posted materials are not copyedited and are the sole responsibility of the authors, so questions or comments should be addressed to the corresponding author.

ciz755_suppl_Supplementary_Figure_1A

ciz755_suppl_Supplementary_Figure_1B-1

ciz755_suppl_Supplementary_Figure_1B-2

ciz755_suppl_Supplementary_Figure_1B-3

ciz755_suppl_Supplementary_Figure_1B-4

ciz755_suppl_Supplementary_Figure_1C-1

ciz755_suppl_Supplementary_Figure_1C-2

ciz755_suppl_Supplementary_Figure_2

ciz755_suppl_Supplementary_Figure_3

ciz755_suppl_Supplementary_Figure_4

ciz755_suppl_Supplementary_Table_1

ciz755_suppl_Supplementary_Table_2

## Notes

***Acknowledgments.*** The authors thank Soo-Young Kwon and Hyon Jin Jeon of the International Vaccine Institute (IVI) for supporting the Typhoid Fever Surveillance in Africa Program (TSAP) in administrative matters. We are grateful for the exceptional efforts of all contributors at the Institut Pasteur de Dakar and associated institutes in Senegal, which made this research possible: household visits/interviewers (Institut Pasteur de Dakar: Adama Dieng, Aliou Faye, Andulaye Diop, Coumba Thiaw, Khadiatou Diop, Mamadou Faye, Majeur Cissé, Souane Moussa); data management (Institut Pasteur de Dakar: Atta Gadji Kandji, Joseph Faye); and scientific support (Diagne Fallou and Lo Ibrahima, Responsable du commune d’arrondisssment de Pikine Ouest; Djibril Diallo Institut Sénégalais de Recherches Agricoles (ISRA), Unité d’Information et de Valorisation (UNIVAL), and Responsable du commune d’arrondisssment de Djiddah Thiaroye Kao; Thiaroye Kao, Unité d’Information et de Valorisation, Responsable du commune d’arrondisssment de Djiddah; Marème Dia Ndiaye, République du Sénégal/Ministère de la Santé et de la Prévention; Souleymane Diop, Centre de Suivi Ecoloquige; Papa Djibril Ba, République du Sénégal/Ministère de l’Economique et des Finances, Agence Nationale de la Statistique et de la Démographie). The authors also thank all contributors at the University of Gezira and the Surgical Pediatric Hospital in Wad-Medani, Sudan, for the exceptional efforts, which allowed the conduct of this research: household visits/interviewers (Iman Gasmelseed, Mohammed Omer, Niama Mubarak, Nuseiba Abdalla, Omnia Adam, Rajaa Bahlol, Rasha Khidir, Suhair Haydar). Finally, the authors are grateful for the exceptional efforts of all contributors at the National Institute for Communicable Diseases (NICD) as well as the Red Cross in South Africa for their outstanding efforts, which made this research possible: household visits/interviewers (Red Cross: Annetta Ndlovu, Bukeka Nzimande, Dumisani Mzobg, Jabu Gumede, Happiness Mthembu, Hlengiwe Ngcobo, Montsikelele Nkwanyana, Ncamsile Yengwa, Nonkululeko Mettunu, Nontobeko Mknize, Sandisa Ndzimela, Sandra Majozi, Peggy Hlubi, Pinky Thabethe, Sbongile Phetha, Siyabonga Hlatshwayo, Sizwe Skosana, Xolani Zulu, Zandile Ndhlovu, Zanele Mncube); data management (NICD: Makatisane Papo, Jabu Mabuyakhulu); administrative support (NICD: Sibongile Walaza); support in study coordination (Red Cross: Derick Naidoo, Zinny Zulu); and scientific support (NICD: Adam Cohen, Cheryl Cohen, Claire von Mollendorf). The study was approved by the respective ethics committees, the Comité National d’Ethique pour la Recherche en Santé in Senegal, the KwaZulu-Natal Provincial Health Research Committee (PHRC) and the Human Research Ethics Committee (Medical) in South Africa, the National Health Research Ethics Committee in Sudan, and the Ethical Review Board of the IVI. Written informed consent was obtained from all study participants.

***Financial support*.** This research was funded by the Bill & Melinda Gates Foundation [OPP1127988]. The International Vaccine Institute acknowledges its donors including the Republic of Korea and the Swedish International Development Cooperation Agency. This publication was made possible through a grant from the Bill & Melinda Gates Foundation [OPP1201031].

***Supplement sponsorship*.** This article was published as part of the supplement “Severe Typhoid Fever in Africa (SETA) Program” sponsored by the International Vaccine Institute.

***Potential conflicts of interest.*** The authors report no potential conflicts of interest. All authors have submitted the ICMJE Form for Disclosure of Potential Conflicts of Interest. Conflicts that the editors consider relevant to the content of the manuscript have been disclosed.
