# Supplementary figures and images for: The Typhoid Fever Surveillance in Africa Program: Geospatial Sampling Frames for Household-based Studies: Lessons Learned From a Multicountry Surveillance Network in Senegal, South Africa, and Sudan
Source: Clin Infect Dis. 2019 Oct 30;69(Suppl 6):S474–82. doi: 10.1093/cid/ciz755 (PMC6821174; doi:10.1093/cid/ciz755)

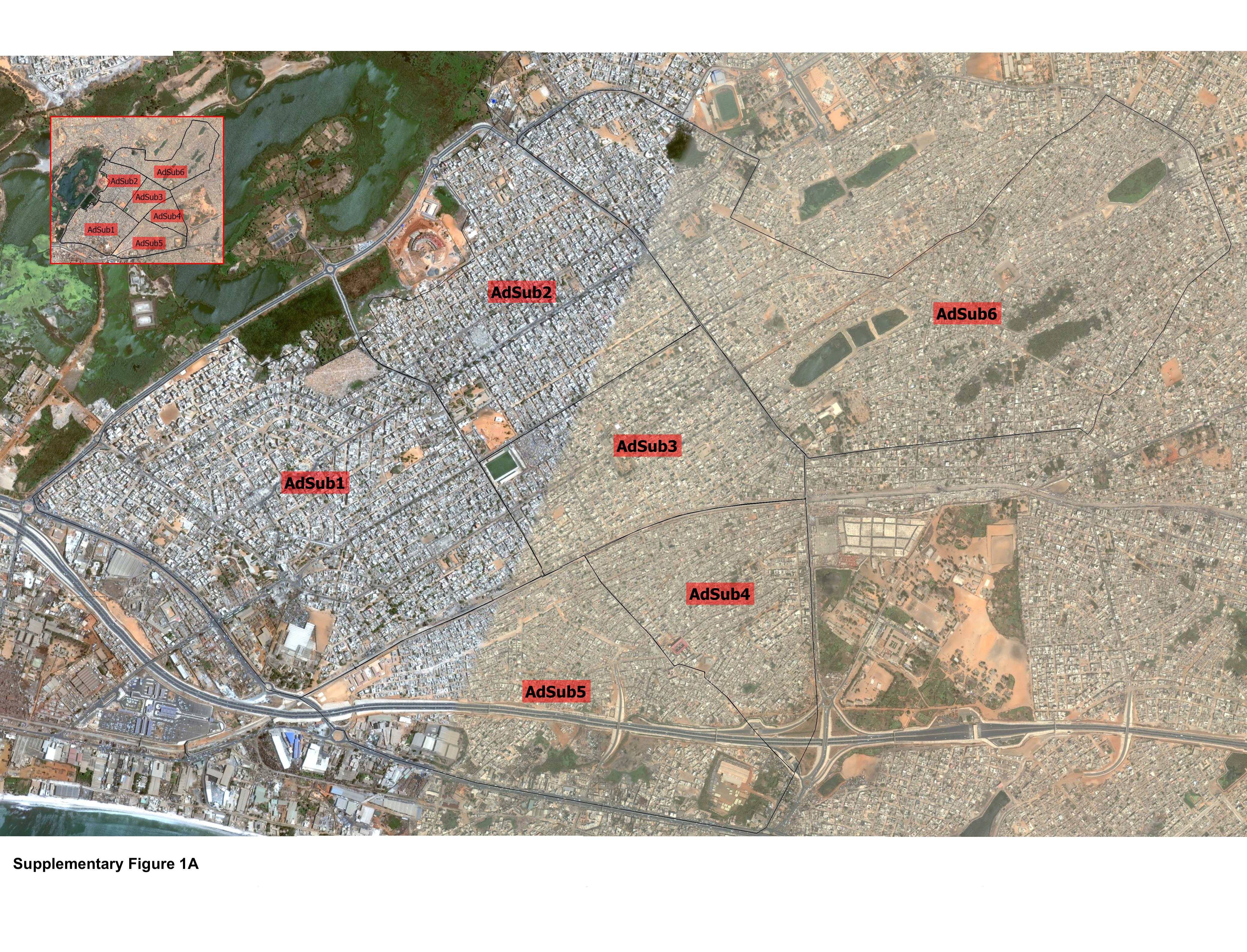

Supplement: ciz755_suppl_Supplementary_Figure_1A [file ciz755_suppl_supplementary_figure_1a.jpeg]

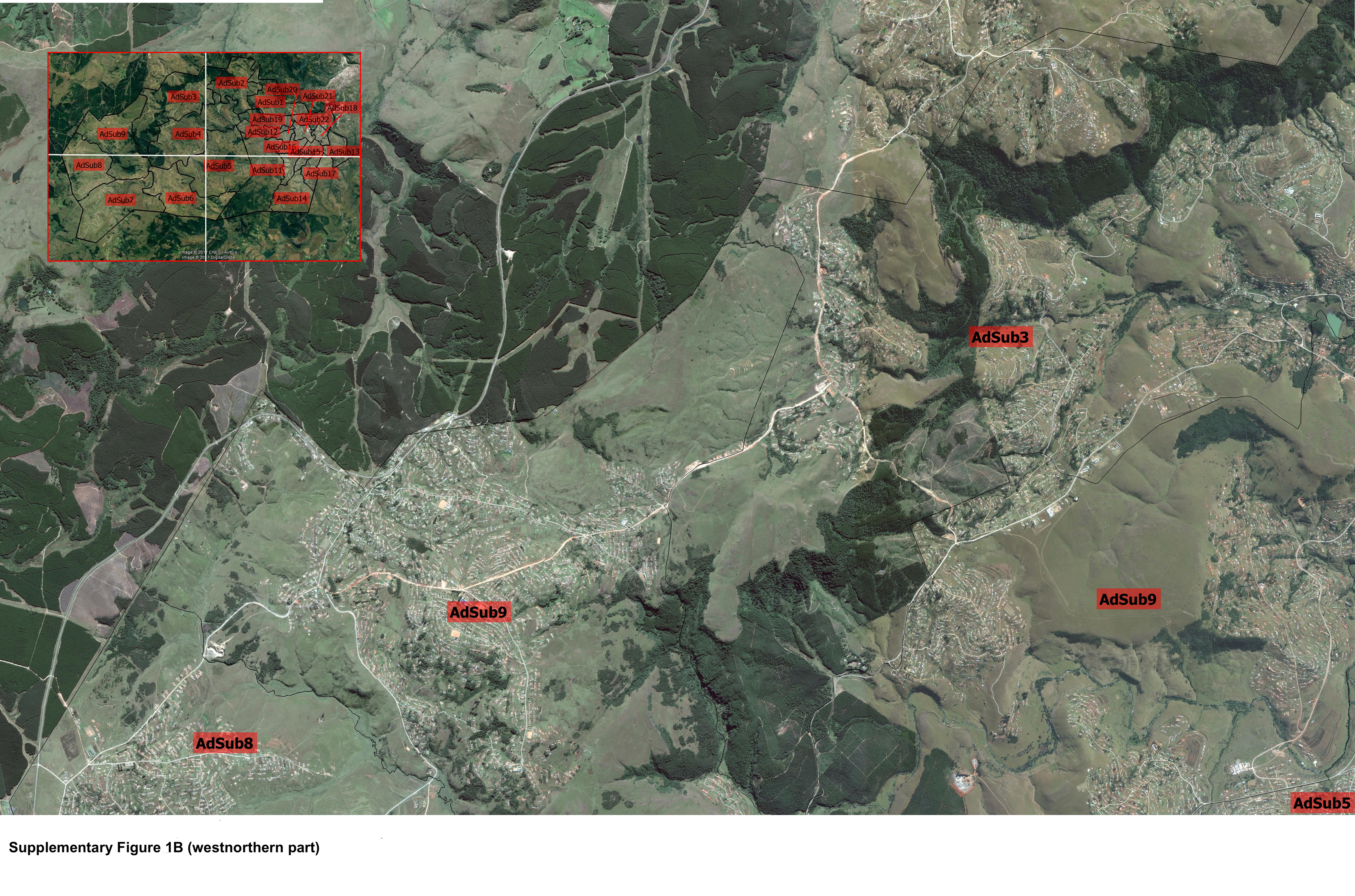

Supplement: ciz755_suppl_Supplementary_Figure_1B-1 [file ciz755_suppl_supplementary_figure_1b-1.jpeg]

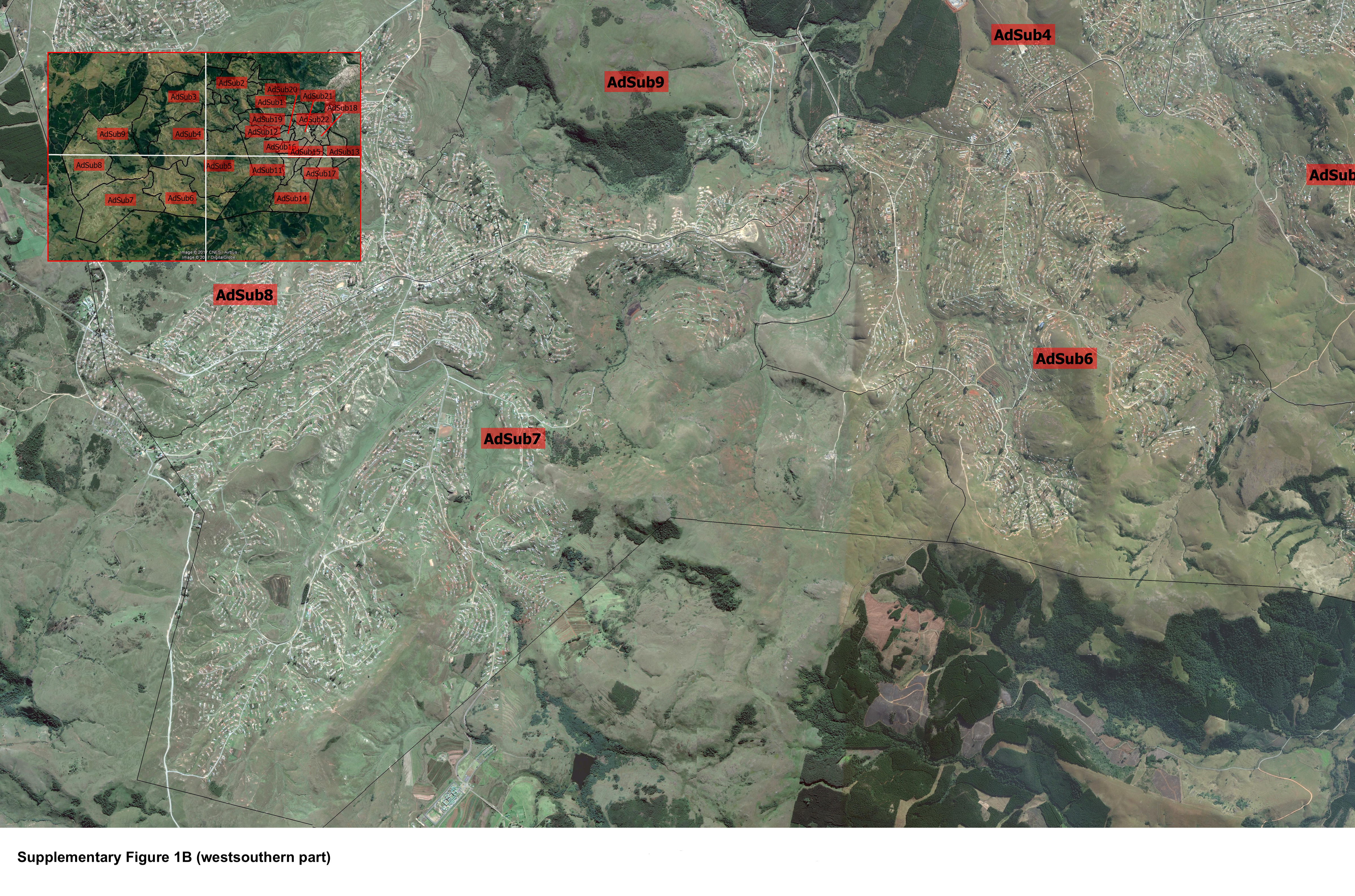

Supplement: ciz755_suppl_Supplementary_Figure_1B-2 [file ciz755_suppl_supplementary_figure_1b-2.jpeg]

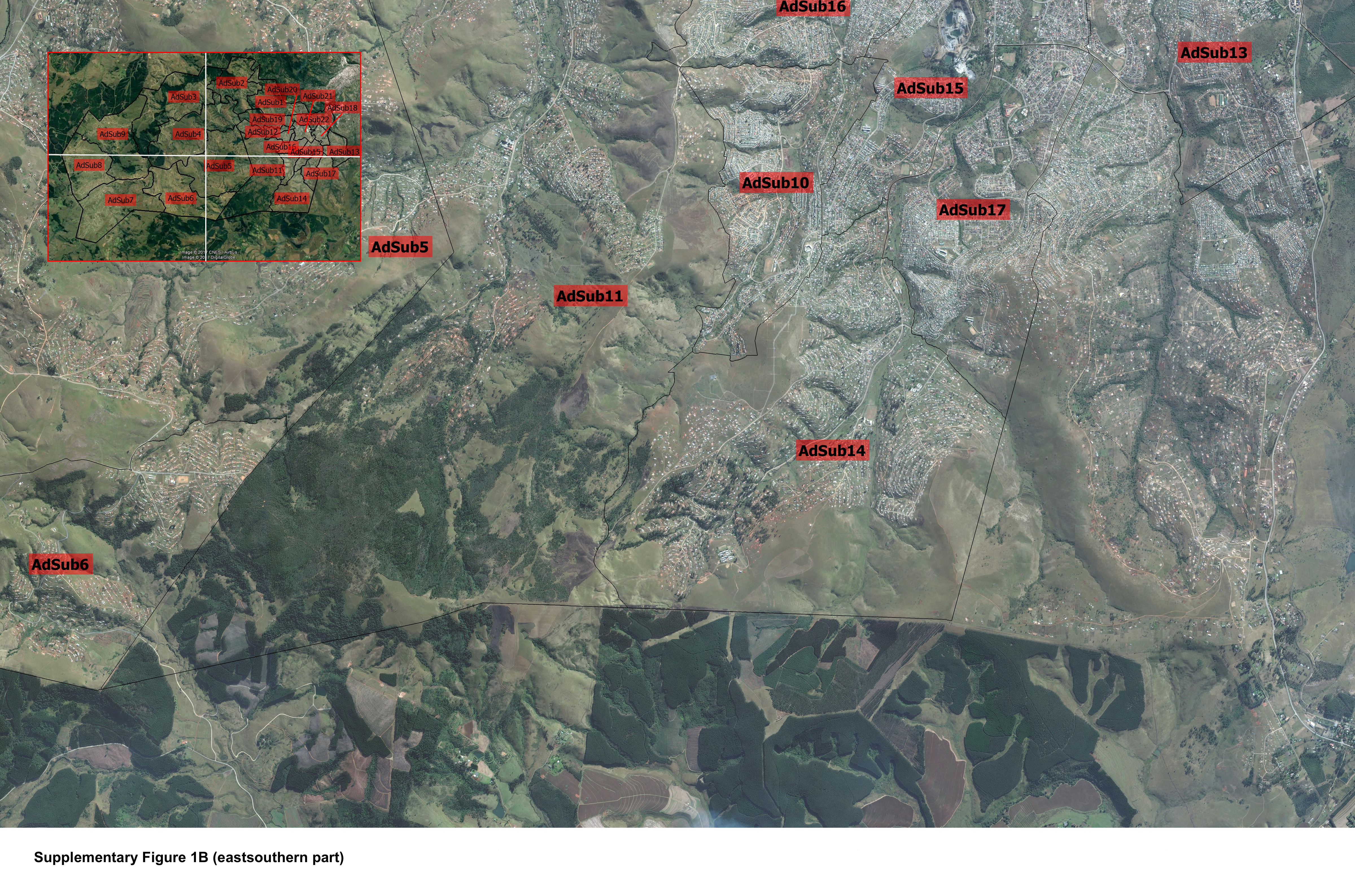

Supplement: ciz755_suppl_Supplementary_Figure_1B-3 [file ciz755_suppl_supplementary_figure_1b-3.jpeg]

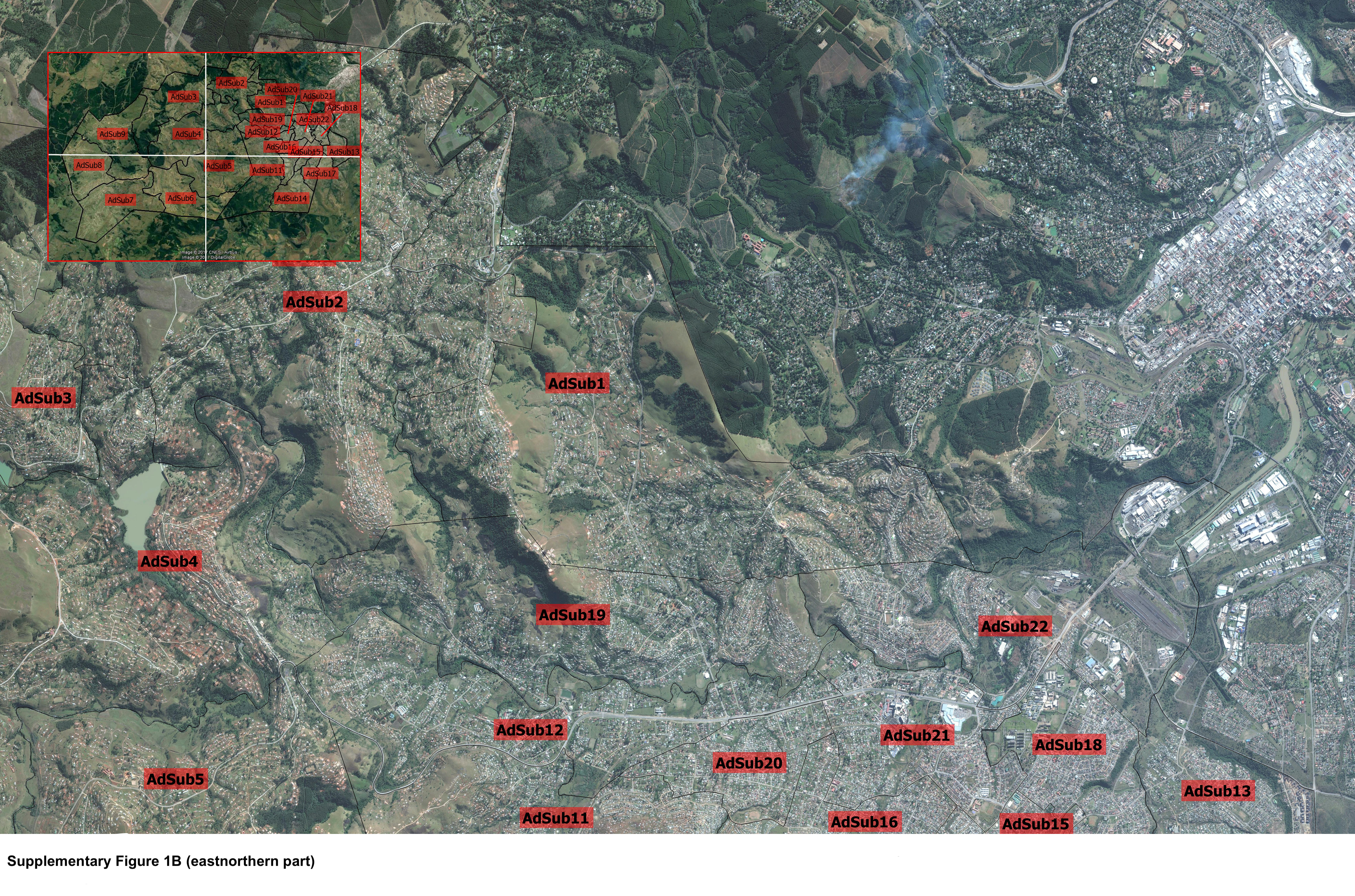

Supplement: ciz755_suppl_Supplementary_Figure_1B-4 [file ciz755_suppl_supplementary_figure_1b-4.jpeg]

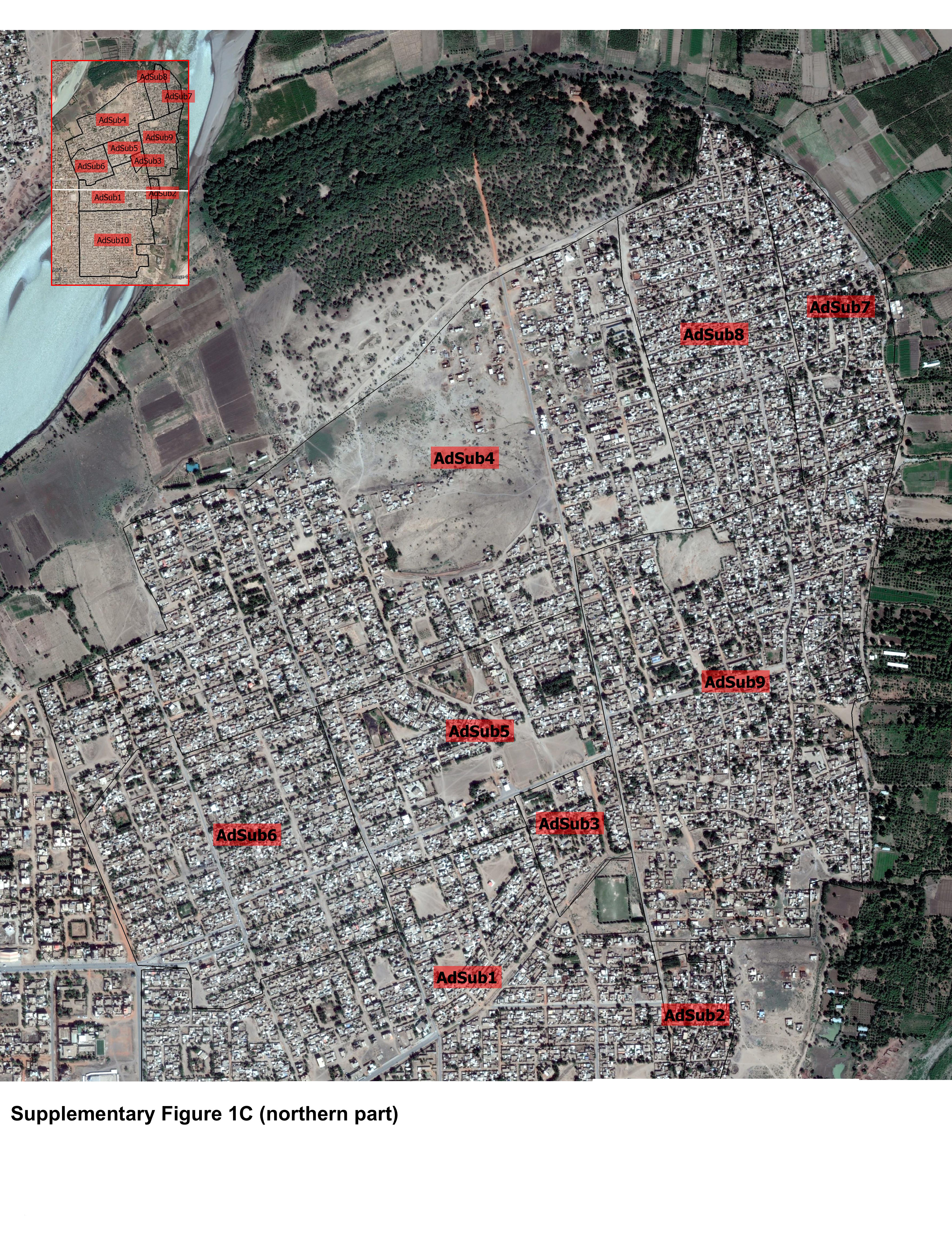

Supplement: ciz755_suppl_Supplementary_Figure_1C-1 [file ciz755_suppl_supplementary_figure_1c-1.jpeg]

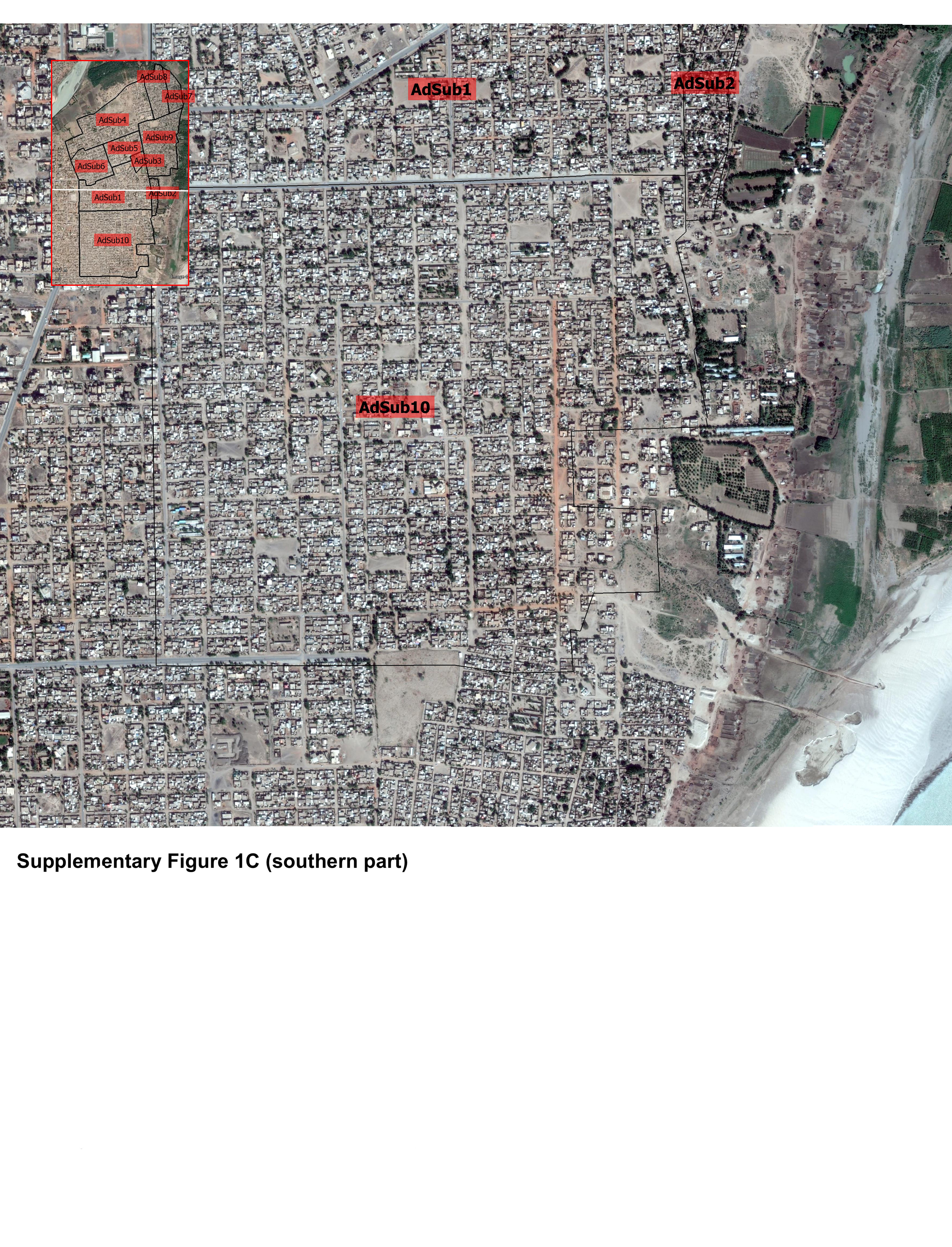

Supplement: ciz755_suppl_Supplementary_Figure_1C-2 [file ciz755_suppl_supplementary_figure_1c-2.jpeg]

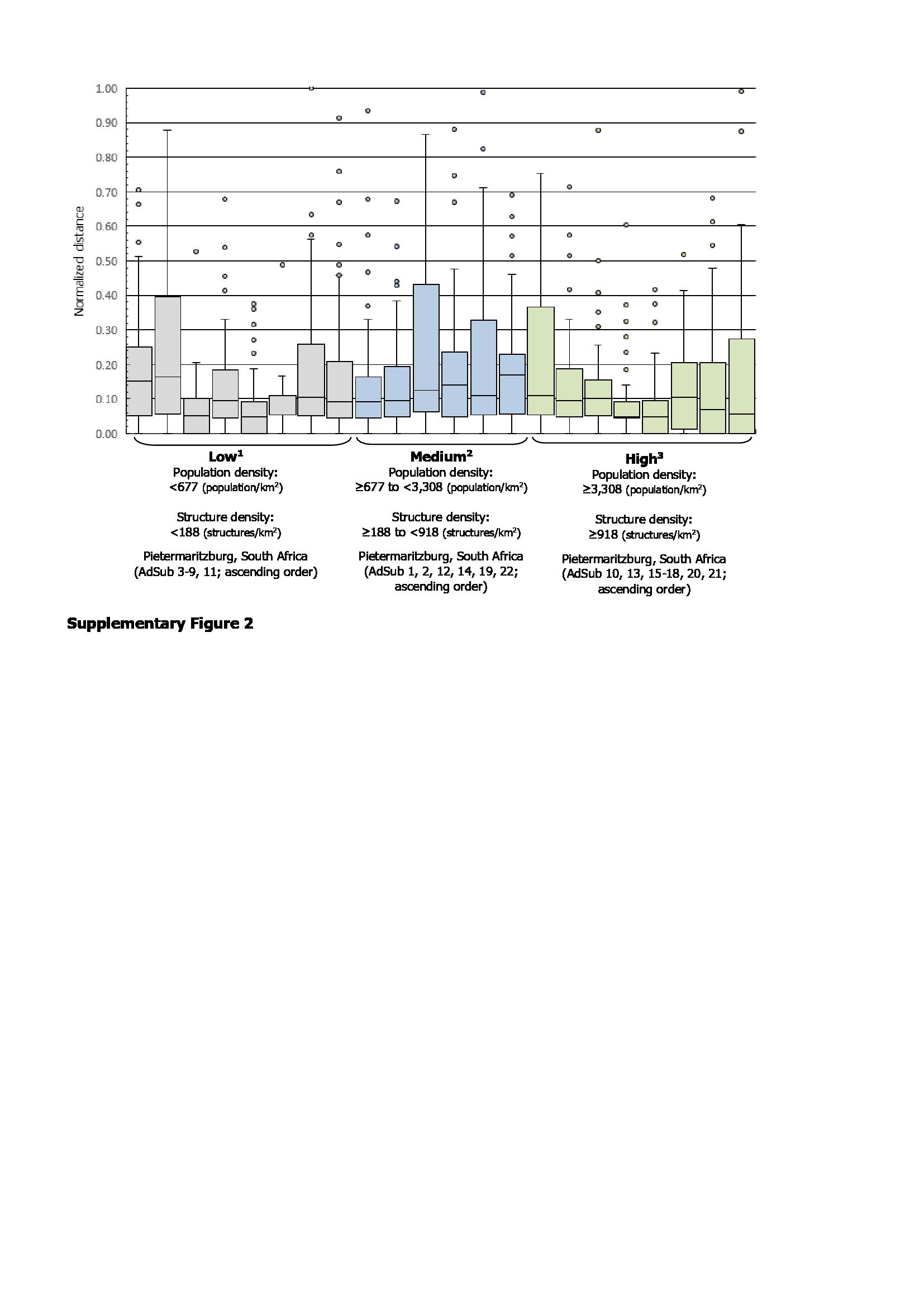

Supplement: ciz755_suppl_Supplementary_Figure_2 [file ciz755_suppl_supplementary_figure_2.jpeg]

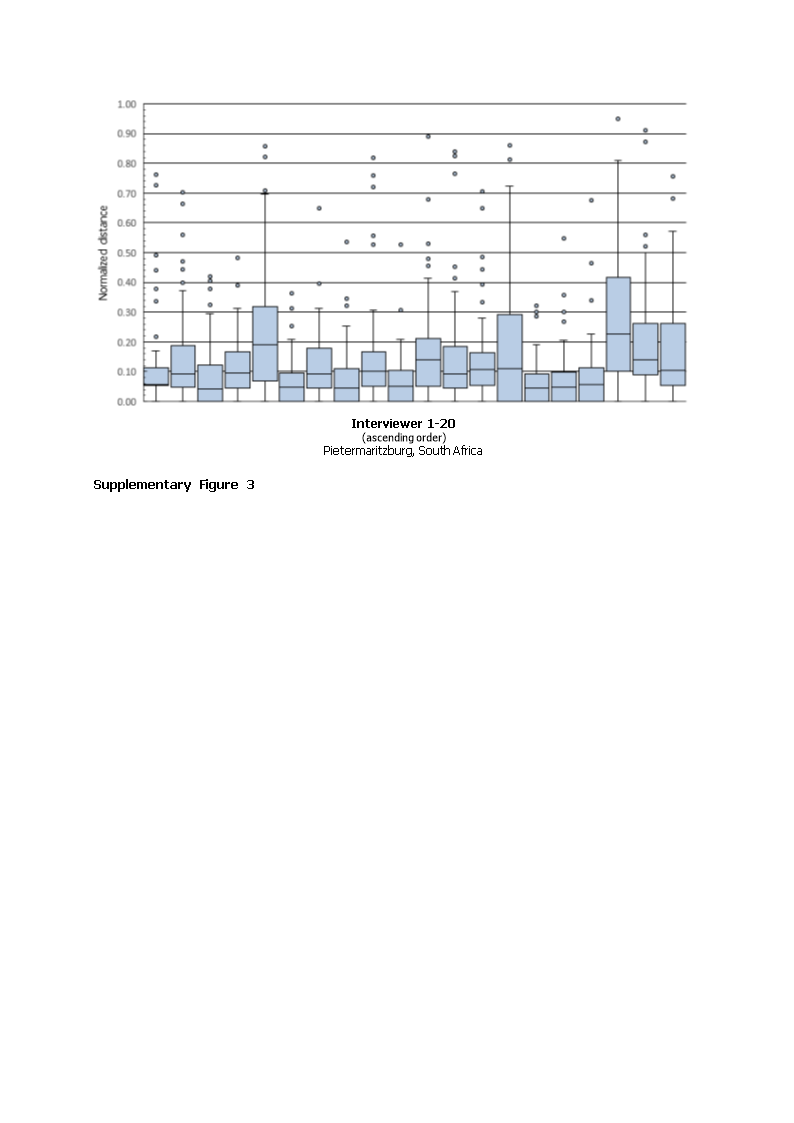

Supplement: ciz755_suppl_Supplementary_Figure_3 [file ciz755_suppl_supplementary_figure_3.png]

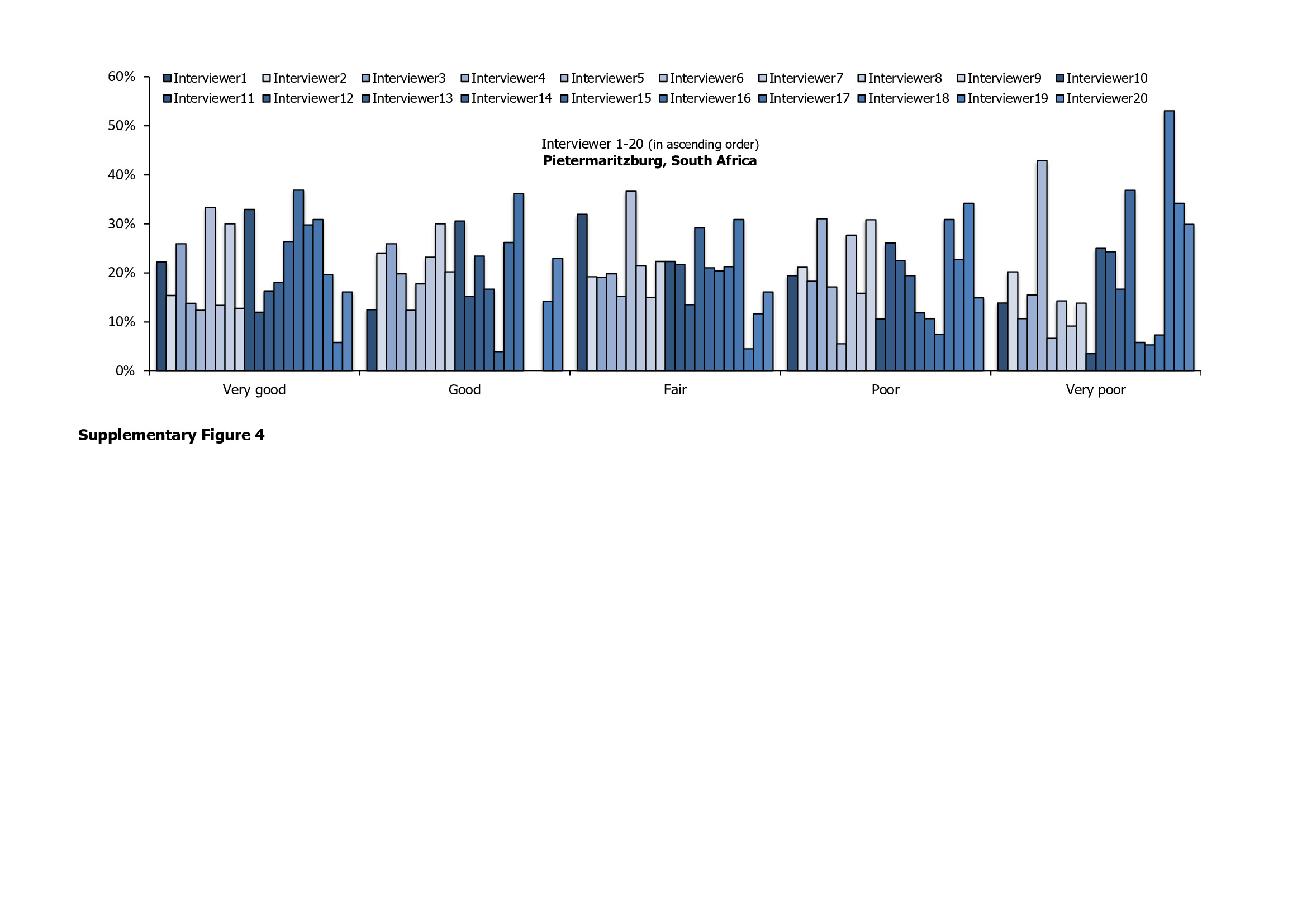

Supplement: ciz755_suppl_Supplementary_Figure_4 [file ciz755_suppl_supplementary_figure_4.jpeg]
