# Supplementary material for: The Typhoid Fever Surveillance in Africa Program: Geospatial Sampling Frames for Household-based Studies: Lessons Learned From a Multicountry Surveillance Network in Senegal, South Africa, and Sudan
Source: Clin Infect Dis. 2019 Oct 30;69(Suppl 6):S474–82. doi: 10.1093/cid/ciz755 (PMC6821174; doi:10.1093/cid/ciz755)
Supplement: ciz755_suppl_Supplementary_Table_1 [file ciz755_suppl_supplementary_table_1.docx]

| Distance calculation between geographic coordinates | | | |
| --- | --- | --- | --- |
| $\boldsymbol{LAT}_{\boldsymbol{diff}}\mathbf{=}\left[ \frac{\boldsymbol{\vert LAT}\boldsymbol{1-LAT}\boldsymbol{2\vert}}{\boldsymbol{360}} \right]\boldsymbol{\cdot PR \cdot2 \cdot}\mathbf{л}$ | | ${LON}_{diff}=\left[ \frac{\left\vert LON1- LON2 \right\vert}{360} \right]\cdot ER \cdot2 \cdotл\cdot\cos\left[ \frac{(LAT1+ LAT2)}{2} \right]$ | |
| Normalized distance (or distance coefficient) between geographic coordinates | | | |
| $\boldsymbol{DIST}_{\boldsymbol{normalized}}\boldsymbol{=}\sqrt{\frac{{\boldsymbol{(LAT}_{\boldsymbol{diff}}\boldsymbol{-}\boldsymbol{LON}_{\boldsymbol{diff}}\boldsymbol{)}}^{\boldsymbol{2}}}{\boldsymbol{N}}}$ | | | |
| Root mean square error (RMSE) of normalized distance | | | |
| $\boldsymbol{RMSE=}\sqrt{\frac{\boldsymbol{1}}{\boldsymbol{N}} \sum{\boldsymbol{(}\boldsymbol{DIST}_{\boldsymbol{coefficient}}\boldsymbol{)}}^{\boldsymbol{2}}}$ | | | |
| Kruskal-Wallis Test (H) and Kruskal-Wallis Test (Hc) applying tie correction, including Bonferroni corrected significance level | | | |
| $\boldsymbol{H=}\frac{\boldsymbol{12}}{\boldsymbol{n (n+1)}}\sum_{\boldsymbol{i=1}}^{\boldsymbol{k}} \frac{{\boldsymbol{R}_{\boldsymbol{i}}}^{\boldsymbol{2}}}{\boldsymbol{n}_{\boldsymbol{i}}}\boldsymbol{-3 (}\mathbf{n+1}\boldsymbol{)}$ | $Hc=\frac{\frac{12}{n (n+1)}\sum_{i=1}^{k} \frac{{R_{i}}^{2}}{n_{i}}-3 (n+1)}{1- \sum_{i=1}^{nd} {{(d}_{i}}^{3}-d_{i})/(n^{3}-n)}$ | | $P=\frac{\alpha}{m}$ |

**Supplementary Table 1** Equations for normalized distance, Root Mean Square Error (RMSE) and Kruskal-Wallis Test.

Equations for calculating the normalized distance between the geographic coordinates of structures taken from the sampling frame and by GPS readings on the ground (based on the Pythagorean Theorem), and the Root Mean Square Error (RMSE) of normalized distances; fromulas for performing the Kruskal-Wallis Test (H) as well as the Kruskal-Wallis Test (Hc) applying tie correction, including Bonferroni corrected significance level.

Abbreviations:

GPS=global positioning system

${LAT}_{diff}$=latitude difference between the satellite image (sampling frame) and the GPS reading (receiver)

$LAT1$=latitude of the structure from the satellite image (sampling frame)

$LAT2$=latitude of the structure obtained from GPS reading (receiver)

$\mathrm{PR}$=polar radius=6,356,750 m

$л$=3.14159

${LON}_{diff}$=longitude difference between the satellite image (sampling frame) and the GPS reading (receiver)

$LON1$=longitude of the structure from the satellite image (sampling frame)

$LON2$=longitude of the structure obtained from GPS reading (receiver)

$\mathrm{ER}$=polar radius=6,378,137 m

${DIST}_{normalized}$=normalized distance

$N$=number of observations

$\mu_{i}$=position of median for sample (i)

$n$=sum of observations of all samples

$n_{i}$=sum of observations of sample (i)

$R_{i}$=sum of ranks of sample (i)

$k$=number of samples studied

$nd$=number of samples with distinct values

$d_{i}$=number of observations for each of the values

$\alpha$=significance level set at 0.05

$P$=corrected significance level

$m$=number of comparisons
