# Supplementary material for: The Typhoid Fever Surveillance in Africa Program: Geospatial Sampling Frames for Household-based Studies: Lessons Learned From a Multicountry Surveillance Network in Senegal, South Africa, and Sudan
Source: Clin Infect Dis. 2019 Oct 30;69(Suppl 6):S474–82. doi: 10.1093/cid/ciz755 (PMC6821174; doi:10.1093/cid/ciz755)
Supplement: ciz755_suppl_Supplementary_Table_2 [file ciz755_suppl_supplementary_table_2.docx]

| Inter  viewer | 1 | 2 | 3 | 4 | 5 | 6 | 7 | 8 | 9 | 10 | 11 | 12 | 13 | 14 | 15 | 16 | 17 | 18 | 19 | 20 |
| --- | --- | --- | --- | --- | --- | --- | --- | --- | --- | --- | --- | --- | --- | --- | --- | --- | --- | --- | --- | --- |
| 1 | 1.0000 | 0.5202 | 0.0097 | 0.7328 | 0.0007 | 0.0042 | 0.7763 | 0.0021 | 0.1505 | 0.0179 | 0.0235 | 0.5273 | 0.4249 | 0.1681 | 0.0006 | 0.0061 | 0.2022 | **0.0003** | 0.0006 | 0.0925 |
| 2 | 0.5202 | 1.0000 | 0.0003 | 0.7258 | 0.0024 | **0.0001** | 0.6827 | **< 0.0001** | 0.3738 | 0.0011 | 0.0716 | 0.9829 | 0.8224 | 0.3960 | **< 0.0001** | **0.0002** | 0.0439 | 0.0009 | 0.0021 | 0.2436 |
| 3 | 0.0097 | 0.0003 | 1.0000 | 0.0007 | **< 0.0001** | 0.5913 | 0.0010 | 0.5310 | **< 0.0001** | 0.9998 | **< 0.0001** | **0.0002** | 0.0005 | **< 0.0001** | 0.2643 | 0.7121 | 0.2734 | **< 0.0001** | **< 0.0001** | **< 0.0001** |
| 4 | 0.7328 | 0.7258 | 0.0007 | 1.0000 | 0.0005 | 0.0003 | 0.9500 | **< 0.0001** | 0.2101 | 0.0026 | 0.0288 | 0.7378 | 0.5857 | 0.2345 | **< 0.0001** | 0.0005 | 0.0806 | **0.0002** | 0.0004 | 0.1264 |
| 5 | 0.0007 | 0.0024 | **< 0.0001** | 0.0005 | 1.0000 | **< 0.0001** | 0.0005 | **< 0.0001** | 0.0384 | **< 0.0001** | 0.2544 | 0.0019 | 0.0116 | 0.0521 | **< 0.0001** | **< 0.0001** | **< 0.0001** | 0.5079 | 0.9434 | 0.0831 |
| 6 | 0.0042 | **0.0001** | 0.5913 | 0.0003 | **< 0.0001** | 1.0000 | 0.0005 | 0.9677 | **< 0.0001** | 0.6268 | **< 0.0001** | **0.0001** | **0.0002** | **< 0.0001** | 0.6105 | 0.8728 | 0.1399 | **< 0.0001** | **< 0.0001** | **< 0.0001** |
| 7 | 0.7763 | 0.6827 | 0.0010 | 0.9500 | 0.0005 | 0.0005 | 1.0000 | **0.0001** | 0.1927 | 0.0033 | 0.0259 | 0.6937 | 0.5510 | 0.2163 | **< 0.0001** | 0.0007 | 0.0926 | **0.0002** | 0.0004 | 0.1153 |
| 8 | 0.0021 | **< 0.0001** | 0.5310 | **< 0.0001** | **< 0.0001** | 0.9677 | **0.0001** | 1.0000 | **< 0.0001** | 0.5765 | **< 0.0001** | **< 0.0001** | **< 0.0001** | **< 0.0001** | 0.6134 | 0.8317 | 0.1096 | **< 0.0001** | **< 0.0001** | **< 0.0001** |
| 9 | 0.1505 | 0.3738 | **< 0.0001** | 0.2101 | 0.0384 | **< 0.0001** | 0.1927 | **< 0.0001** | 1.0000 | **< 0.0001** | 0.3706 | 0.3556 | 0.5562 | 0.9922 | **< 0.0001** | **< 0.0001** | 0.0056 | 0.0132 | 0.0388 | 0.7733 |
| 10 | 0.0179 | 0.0011 | 0.9998 | 0.0026 | **< 0.0001** | 0.6268 | 0.0033 | 0.5765 | **< 0.0001** | 1.0000 | **< 0.0001** | 0.0010 | 0.0014 | **0.0001** | 0.3157 | 0.7388 | 0.3144 | **< 0.0001** | **< 0.0001** | **< 0.0001** |
| 11 | 0.0235 | 0.0716 | **< 0.0001** | 0.0288 | 0.2544 | **< 0.0001** | 0.0259 | **< 0.0001** | 0.3706 | **< 0.0001** | 1.0000 | 0.0644 | 0.1556 | 0.4024 | **< 0.0001** | **< 0.0001** | 0.0003 | 0.0982 | 0.2687 | 0.5542 |
| 12 | 0.5273 | 0.9829 | **0.0002** | 0.7378 | 0.0019 | **0.0001** | 0.6937 | **< 0.0001** | 0.3556 | 0.0010 | 0.0644 | 1.0000 | 0.8051 | 0.3789 | **< 0.0001** | **0.0002** | 0.0432 | 0.0007 | 0.0017 | 0.2288 |
| 13 | 0.4249 | 0.8224 | 0.0005 | 0.5857 | 0.0116 | **0.0002** | 0.5510 | **< 0.0001** | 0.5562 | 0.0014 | 0.1556 | 0.8051 | 1.0000 | 0.5689 | **< 0.0001** | 0.0003 | 0.0392 | 0.0040 | 0.0115 | 0.3968 |
| 14 | 0.1681 | 0.3960 | **< 0.0001** | 0.2345 | 0.0521 | **< 0.0001** | 0.2163 | **< 0.0001** | 0.9922 | **0.0001** | 0.4024 | 0.3789 | 0.5689 | 1.0000 | **< 0.0001** | **< 0.0001** | 0.0081 | 0.0184 | 0.0535 | 0.7924 |
| 15 | 0.0006 | **< 0.0001** | 0.2643 | **< 0.0001** | **< 0.0001** | 0.6105 | **< 0.0001** | 0.6134 | **< 0.0001** | 0.3157 | **< 0.0001** | **< 0.0001** | **< 0.0001** | **< 0.0001** | 1.0000 | 0.4960 | 0.0468 | **< 0.0001** | **< 0.0001** | **< 0.0001** |
| 16 | 0.0061 | **0.0002** | 0.7121 | 0.0005 | **< 0.0001** | 0.8728 | 0.0007 | 0.8317 | **< 0.0001** | 0.7388 | **< 0.0001** | **0.0002** | 0.0003 | **< 0.0001** | 0.4960 | 1.0000 | 0.1797 | **< 0.0001** | **< 0.0001** | **< 0.0001** |
| 17 | 0.2022 | 0.0439 | 0.2734 | 0.0806 | **< 0.0001** | 0.1399 | 0.0926 | 0.1096 | 0.0056 | 0.3144 | 0.0003 | 0.0432 | 0.0392 | 0.0081 | 0.0468 | 0.1797 | 1.0000 | **< 0.0001** | **< 0.0001** | 0.0028 |
| 18 | **0.0003** | 0.0009 | **< 0.0001** | **0.0002** | 0.5079 | **< 0.0001** | **0.0002** | **< 0.0001** | 0.0132 | **< 0.0001** | 0.0982 | 0.0007 | 0.0040 | 0.0184 | **< 0.0001** | **< 0.0001** | **< 0.0001** | 1.0000 | 0.4590 | 0.0295 |
| 19 | 0.0006 | 0.0021 | **< 0.0001** | 0.0004 | 0.9434 | **< 0.0001** | 0.0004 | **< 0.0001** | 0.0388 | **< 0.0001** | 0.2687 | 0.0017 | 0.0115 | 0.0535 | **< 0.0001** | **< 0.0001** | **< 0.0001** | 0.4590 | 1.0000 | 0.0860 |
| 20 | 0.0925 | 0.2436 | **< 0.0001** | 0.1264 | 0.0831 | **< 0.0001** | 0.1153 | **< 0.0001** | 0.7733 | **< 0.0001** | 0.5542 | 0.2288 | 0.3968 | 0.7924 | **< 0.0001** | **< 0.0001** | 0.0028 | 0.0295 | 0.0860 | 1.0000 |

Bonferroni corrected significance level: 0.0003

**Supplementary Table 2** *p*-values of Dunn’s multiple pairwise comparisons of normalized distances by interviewer in Pietermaritzburg, South Africa.

Two-tailed *p*-values significant (bold) and non-significant (regular) are shown; the intersecting sets of significant *p*-values of interviewers that located structures most (dark grey rectangles) and least (light grey rectangles) accurately are illustrated.
